# A Rare NPHS2 Mutation (E130K) in Hereditary Steroid‐Resistant Nephrotic Syndrome: A Case Report

**DOI:** 10.1155/crin/8289305

**Published:** 2026-01-29

**Authors:** Ramzi Hmedan Mujahed, Omar Hammam Salloum, Nimatallah Fares Ishreiteh, Hiba Mofdi Hosheyh, Aisha Dahood Abu Hashem, Yara Fadi Shamestti, Malak Ramzy Hroub

**Affiliations:** ^1^ Department of Pediatrics, Hebron Governmental Hospital, Hebron, State of Palestine; ^2^ Faculty of Medicine, Palestine Polytechnic University, Hebron, State of Palestine, ppu.edu; ^3^ Faculty of Medicine, Al-Quds University, East Jerusalem, State of Palestine, alquds.edu

**Keywords:** congenital nephrotic syndrome, focal segmental glomerulosclerosis (FSGS), genetic nephropathy, hereditary kidney disease, minimal change disease (MCD), NPHS2 mutation, pediatric nephrology, podocin, proteinuria, steroid-resistant nephrotic syndrome (SRNS)

## Abstract

Hereditary steroid‐resistant nephrotic syndrome (HSRNS) due to mutations in the NPHS2 gene (encoding podocin) is a rare genetic condition that typically presents in childhood. We report a case of a 2‐year‐and‐8‐month‐old male, the seventh child of consanguineous parents, who presented with recurrent fever, febrile tonic‐clonic seizures, and periorbital edema. His medical history included multiple hospitalizations in infancy due to suspected sepsis and chest infections. Upon admission, laboratory findings revealed proteinuria, hypoalbuminemia, and hypercholesterolemia, along with mild hepatomegaly. Genetic testing identified compound heterozygous mutations (R138X and E130K) in the NPHS2 gene, confirming a diagnosis of HSRNS. Renal biopsy revealed features consistent with minimal change disease, and immunofluorescence was negative for IgG, IgM, IgA, C3, and C4. The patient was treated with enalapril as part of supportive management. This case highlights the importance of early genetic testing and renal biopsy in diagnosing steroid‐resistant nephrotic syndrome, particularly in children with atypical presentations. Understanding the genetic underpinnings of such rare cases is essential for guiding appropriate treatment and providing prognostic insights.

## 1. Introduction

Congenital nephrotic syndrome (CNS) is a rare kidney disorder that leads to severe protein loss in the urine and swelling (edema), usually appearing before birth or within the first 3 months of life [1]. While some cases are caused by infections or maternal immune conditions, most result from genetic mutations that disrupt podocytes, the specialized kidney cells responsible for filtration [1]. This disruption weakens the kidney’s ability to retain essential proteins, leading to progressive kidney damage [1].

Children with CNS often experience generalized edema, low albumin levels (hypoalbuminemia), significant proteinuria, and dyslipidemia early in life [2]. Without timely intervention, it can progress to end‐stage kidney disease (ESKD), requiring long‐term renal support [2].

Nephrotic syndrome (NS) is a common kidney condition marked by heavy proteinuria [1, 3]. While most cases occur randomly, about 3%–5% are inherited [3]. Mutations in NPHS1, NPHS2, ACTN4, CD2AP, WT1, TRPC6, and LAMB2 affect crucial proteins like nephrin, podocin, alpha‐actinin‐4, and CD2‐associated protein, all of which help maintain the kidney’s filtration barrier. When these proteins are defective, excessive protein loss occurs, leading to chronic kidney complications [3].

Genetic testing has transformed the way CNS is diagnosed and managed [4]. It helps doctors tailor treatments to each child, avoid unnecessary steroid and immunosuppressive therapy, predict how the disease will progress, provide prenatal counseling for families, and deepen the overall understanding of its genetic causes [4].

Here, we present a rare case of hereditary steroid‐resistant nephrotic syndrome (HSRNS) in a 2‐year‐old child caused by compound heterozygous mutations in the NPHS2 gene. The child initially presented with febrile seizures and periorbital edema, highlighting an atypical clinical manifestation. This case underscores the importance of genetic testing in diagnosing and managing childhood NS.

## 2. Case Presentation

A 2‐year‐and‐8‐month‐old Arabic Palestinian male, the seventh child of consanguineous (paternal cousins) parents, was admitted to the hospital following a series of febrile tonic‐clonic seizures. These seizures, characterized by frothy oral secretions, lasted for only a few minutes and resolved spontaneously without medication. The patient had a history of recurrent fever (39°C axillary) and multiple hospitalizations in infancy: one at 13 days of age for suspected sepsis and another at 2 months for chest infections. Despite these early challenges, the child remained in good health until the age of 2 years and 8 months, when he developed his first episode of febrile convulsions, accompanied by periorbital edema. No history of lower or upper extremity edema, abdominal pain, nausea, or vomiting was noted.

On admission, the patient presented with a temperature of 39°C (axillary), a heart rate of 130 bpm, blood pressure of 113/59 mmHg, and an oxygen saturation of 98%. Hepatomegaly was detected during the physical examination, which was confirmed by ultrasound. Periorbital edema was evident, but there were no signs of generalized edema.

Given the unusual presentation of febrile seizures associated with periorbital edema, a nephrological workup was initiated. Laboratory investigations revealed significant findings of proteinuria, hypoalbuminemia, and hypercholesterolemia, raising concern for an underlying renal condition (Table 1). His white blood cell count and platelet count were elevated, and his erythrocyte sedimentation rate (ESR) was markedly increased (125 mm/hr). Urine analysis revealed proteinuria with a 24‐h collection rate of 50 mg/mL/hr. Serum tests showed a low albumin level (2.1 g/dL), and cholesterol levels were elevated at 380 mg/dL (Table 2).

**TABLE 1:** Patient’s laboratory findings at initial presentation.

Test	Result	Normal range
Hemoglobin (Hb)	12.9 g/dL	11.5–13.5 g/dL
White blood cells (WBC)	11.4 × 10^3^/μL	5.0–15.0 × 10^3^/μL
Platelet count	535 × 10^3^/μL	150–450 × 10^3^/μL
ESR	125 mm/hr	0–10 mm/hr
CRP	Negative	Negative
Sodium (Na)	133 mEq/L	135–145 mEq/L
Potassium (K)	4.1 mEq/L	3.5–5.0 mEq/L
Calcium (Ca)	8.7 mg/dL	8.8–10.8 mg/dL
Total protein	4.7 g/dL	6.0–8.0 g/dL
Albumin	2.1 g/dL	3.5–5.5 g/dL
Cholesterol	380 mg/dL	< 170 mg/dL
Triglycerides	227 mg/dL	< 150 mg/dL

*Note:* This table summarizes the patient’s laboratory results upon hospital admission, highlighting abnormalities consistent with nephrotic syndrome, including proteinuria, hypoalbuminemia, hypercholesterolemia, and elevated inflammatory markers.

**TABLE 2:** Urinalysis results.

Parameter	Result	Normal range
Specific gravity	1.025	1.005–1.030
pH	Acidic	4.5–8
White blood cells (WBCs)	0–2 per HPF	0–5 per HPF
Red blood cells (RBCs)	1–3 per HPF	0–2 per HPF
24 hr protein collection	50 mg/mL/hr	< 100 mg/m^2^/day

*Note:* Urinalysis findings demonstrating significant proteinuria, acidic pH, and slightly increased red blood cells, supporting the diagnosis of nephrotic syndrome.

Genetic testing was performed, revealing compound heterozygous mutations in the NPHS2 gene (encoding the podocin protein) (Table 3). The patient was found to carry the following:

• R138X mutation: documented in previous studies involving familial idiopathic NS (Nat Genet. 2000 April 4 (4): 349–354).
• E130K polymorphism: a rare variant documented in limited population including a Palestinian cohort study (Fadel A. Sharif et al. 2017 December 1: 1, 27–30). However, the pathogenicity and the clinical significance of this variation are unclear. The clinical significance of this variation is unclear, and clinical correlation is recommended. Using the PredictSNP software, the substitution at codon 130 was predicted to have a 76% deleterious effect on the protein function of podocin.

**TABLE 3:** Genetic findings of NPHS2 mutation.

Gene	Mutation	Exon	Nucleotide change	Predicted effect	Reference
NPHS2	E130K	3	c.388G>A	Substitution of glutamic acid to lysine in podocin protein	Novel
NPHS2	R138X	3	c.412C>T	Early stop codon leading to truncated podocin	Previously documented

*Note:* Summary of the genetic sequencing results indicating compound heterozygous mutations (E130K and R138X) in exon 3 of the NPHS2 gene.

Note: Segregation studies involving other family members are indicated to understand the inheritance pattern and clinical significance of the E130K variation.

Renal biopsy showed features consistent with minimal change disease, including focal segmental mesangial changes without interstitial fibrosis or segmental glomerulosclerosis (Table 4). Immunofluorescence staining for IgG, IgM, IgA, C3, and C4 was negative, supporting the diagnosis of minimal change disease, which typically responds to steroids but remains resistant in hereditary cases like this one.

**TABLE 4:** Histopathological and immunofluorescence findings.

Parameter	Finding
Glomeruli examined	24 (none sclerotic)
Sclerosis	Absent
Mesangial cellular increase	Focal, segmental
Tubular injury	Present (mild)
Interstitial fibrosis	Absent
C3 and C4 deposition	Negative
IgG, IgM, IgA	Negative
Podocyte foot process effacement	Present

*Note:* Renal biopsy findings demonstrating minimal change disease (MCD) with no immune complex deposition, supporting the diagnosis of congenital nephrotic syndrome.

The patient was started on enalapril (5 mg orally once daily) as part of supportive treatment. Over the following weeks, the patient responded well to therapy, with a noticeable reduction in proteinuria and periorbital edema. No significant complications such as infections or thromboembolic events were noted during the course of treatment. His renal function remained stable, and follow‐up visits will continue to monitor his condition and ensure appropriate management of the nephrotic syndrome. As the condition is genetically driven, the focus of treatment remains symptomatic, with the aim of reducing protein loss and maintaining renal function.

### 2.1. Differential Diagnosis

Given the patient’s clinical presentation of febrile tonic‐clonic seizures, periorbital edema, and proteinuria, several conditions were initially considered. However, through a combination of clinical evaluation, laboratory testing, and imaging, the following differential diagnoses were carefully excluded (Table 5).

**TABLE 5:** Differential diagnosis and rationale for exclusion.

Differential diagnosis	Key features	Reason for exclusion
Meningitis	Fever, seizures, irritability, altered mental status	Normal EEG, no meningeal irritation, negative CRP
Encephalitis	Fever, seizures, altered consciousness, neurological deficits	No focal neurological signs, normal EEG, no CSF pleocytosis
Orbital cellulitis	Periorbital edema, fever, erythema, proptosis, pain with eye movement	No eyelid redness, no ocular motility restriction, normal CT scan
Metabolic disorders	Episodic seizures, metabolic acidosis, hyperammonemia, failure to thrive	Normal electrolytes, anion gap, no metabolic acidosis
Autoimmune disorders (e.g., SLE)	Fever, seizures, periorbital edema, nephritis	Normal C3/C4, negative autoantibodies, no systemic lupus criteria met
Hereditary steroid‐resistant nephrotic syndrome (confirmed diagnosis)	Proteinuria, hypoalbuminemia, hypercholesterolemia, periorbital edema, minimal change disease on renal biopsy	Confirmed by genetic testing (NPHS2 mutations E130K and R138X), resistance to steroids, and renal biopsy findings

*Note:* This table summarizes possible differential diagnoses for the patient’s presentation and explains how each was excluded based on clinical, laboratory, and imaging findings.

## 3. Discussion

In this case, we describe a child with CNS who carries compound heterozygous mutations (R138X and E130K) in exon 3 of the NPHS2 gene.

NS is the most common kidney disease in children, affecting 2–7 per 100,000 children worldwide [5]. While most cases occur sporadically, about 3%–5% are inherited [6]. Several genes have been linked to more severe forms of NS, including NPHS1, NPHS2, ACTN4, CD2AP, WT1, TRPC6, and LAMB2, with NPHS2 being one of the most commonly associated with steroid‐resistant nephrotic syndrome (SRNS) [7].

A review of genetic studies shows that NPHS1 mutations account for 15% of CNS cases in Asian populations, while NPHS2 mutations are found in about 11% [8]. The PodoNet Registry also reports that 12.6% of SRNS and CNS patients have NPHS1 mutations, reinforcing the role of podocin and nephrin in the development of NS [9].

### 3.1. Genetic Findings in This Case

Mutations in NPHS2, which encodes podocin, are one of the most well‐documented causes of autosomal recessive SRNS, contributing to 30% of sporadic cases and 20%–40% of familial cases [10]. Over the years, more than 100 different NPHS2 mutations have been reported, affecting podocin’s structure and function [3, 11].

Our patient was found to have a compound heterozygous mutation in NPHS2 (R138X and E130K):

• E130K mutation: a G‐to‐A substitution in exon 3 at position 388, which replaces glutamic acid with lysine in the podocin protein. Since these two amino acids have different properties, this substitution likely alters podocin’s function. The mutation occurs in the prohibitin homology (PHB) domain, a critical region for podocin’s interaction with nephrin, which is essential for kidney filtration [12]. Given that E130K has not been previously reported, functional studies are needed to determine its precise impact on podocin stability and interaction with nephrin. Such investigations could provide valuable insights into its role in disease progression.
• R138X mutation: first described by Boute et al. [13, 14], this C‐to‐T substitution at position 412 in exon 3 introduces an early stop codon, leading to a truncated and nonfunctional podocin protein. This severely affects podocyte function and contributes to steroid resistance in nephrotic syndrome.

Since genetic testing of the parents was not performed, we could not confirm whether these mutations are located on the same allele (cis) or opposite alleles (trans), which is a limitation of this study.

### 3.2. Atypical Histopathology in This Case

Most cases of SRNS caused by NPHS2 mutations are associated with focal segmental glomerulosclerosis (FSGS). However, in our patient, renal biopsy revealed minimal change disease (MCD)—a finding more commonly linked to steroid‐sensitive nephrotic syndrome (SSNS) and NPHS1 mutations [15]. This discrepancy suggests that NPHS2‐related disease may present with more histological variation than previously thought, emphasizing the need for further research.

### 3.3. Genetic Variability in Different Populations

Several studies have examined NPHS2 mutations in different populations:

• A Palestinian study found NPHS2 mutations in 15% of familial SRNS cases (3 out of 20 patients), including R138X, G130K, and R229Q [16].
• In a Saudi Arabian cohort, 44% of familial SRNS patients had NPHS2 mutations, with some carrying previously undescribed variants (p.Q39X and p.R138P) [17].
• An Egyptian cross‐sectional study of 16 CNS patients identified five NPHS2 mutations, including two homozygous (P43Afs∗64 and P20L) and three novel heterozygous missense mutations (M115I, T116S, and D244E) [18].

Published data on the E130K mutation are very limited; one cohort on Palestinian children with SRNS mentioned it [15], nevertheless, further research is needed to understand its effect on protein function and disease progression.

### 3.4. Importance of Genetic Testing in SRNS

Since NPHS2 mutations often indicate steroid resistance, early genetic testing plays a crucial role in avoiding unnecessary exposure to corticosteroids and immunosuppressants. Identifying these mutations allows clinicians to focus on supportive therapies such as ACE inhibitors, which have been shown to reduce proteinuria and slow disease progression [19].

### 3.5. Long‐Term Prognosis and Risk of Progression

Children with NPHS2 mutations are at high risk of disease progression. Regular monitoring for proteinuria, declining kidney function, and hypertension is crucial, as many patients eventually develop ESKD. In such cases, renal transplantation remains the definitive treatment [19].

### 3.6. Potential Treatment Advancements

While current management focuses on supportive therapy, emerging research in podocyte‐targeted treatments and gene therapy may provide new therapeutic options in the future. Advances in gene‐editing techniques, such as CRISPR‐based approaches, hold promise for correcting genetic mutations at their source, potentially altering the disease course for hereditary NS.

## 4. Conclusion

We identified compound heterozygous NPHS2 mutations (E130K and R138X) in a child with HSRNS. Since there are few published studies on E130K, it remains a rare NPHS2 variant with limited and unclear data on its pathogenicity.

Further functional studies are needed to determine its pathogenicity and to better understand how E130K and R138X together influence kidney function, histology, and long‐term prognosis.

## Author Contributions

Ramzi Hmedan Mujahed: supervised the study, provided clinical expertise, and contributed to manuscript revision and critical review. Omar Hammam Salloum: conceptualized the study, led manuscript writing, coordinated data collection, and managed correspondence with the journal. Nimatallah Fares Ishreiteh: conducted data acquisition, assisted in literature review, and contributed to manuscript drafting. Hiba Mofdi Hosheyh: analyzed patient data, contributed to result interpretation, and participated in manuscript revision. Aisha Dahood Abu Hashem: conducted literature review, assisted in manuscript structuring, and contributed to discussion formulation. Yara Fadi Shamestti: assisted in manuscript refinement, proofread for clarity and coherence, and contributed to referencing and formatting. Malak Ramzy Hroub: contributed to reviewing and editing the final draft and submission of the manuscript.

## Funding

No funding was received for this research.

## Disclosure

All authors have read and approved the final manuscript and agree to be accountable for all aspects of the work, ensuring accuracy and integrity in its content.

## Ethics Statement

This case report did not require review by an Institutional Ethics Committee. Written informed consent was obtained from the patient’s legal guardian for the publication of this case report and any accompanying images. A copy of the written consent is available for review by the editor upon request.

## Conflicts of Interest

The authors declare no conflicts of interest.

## Data Availability

Data sharing is not applicable to this article as no datasets were generated or analyzed during the current study.
